# Prognostic value of excision repair cross-complementation group 1 expression in gastric cancer: A meta-analysis

**DOI:** 10.3892/etm.2015.2284

**Published:** 2015-02-11

**Authors:** PENG SONG, QIN YIN, MING LU, BO FU, BAOLIN WANG, QINGHONG ZHAO

**Affiliations:** 1 Department of General Surgery, The Second Affiliated Hospital of Nanjing Medical University, Nanjing, Jiangsu 210011, P.R. China; 2 Medical Center of Pediatrics, The Second Affiliated Hospital of Nanjing Medical University, Nanjing, Jiangsu 210011, P.R. China

**Keywords:** excision repair cross-complementation group 1, gastric cancer, meta-analysis

## Abstract

The prognostic impact of excision repair cross-complementation group 1 (ERCC1) expression in gastric cancer (GC) has been investigated for decades, but has yielded controversial results. The aim of the present study was to provide a precise evaluation of whether the expression levels of ERCC1 are associated with overall survival (OS) in patients with GC. A systematic search of Medline and Embase was conducted. Original studies concerning OS and ERCC1 expression were included for critical appraisal. A total of 15 studies comprising 1,425 patients with GC were identified. The results revealed that high/positive ERCC1 expression was an indicator of poor survival in patients with GC [hazard ratio (HR) 1.48; 95% confidence interval (CI) 1.02–2.10; P=0.036; I^2^=83.8%; random-effects model] compared with low/negative ERCC1 expression. Subgroup analysis indicated that high/positive ERCC1 expression had a significant unfavorable impact on OS in the group of patients evaluated by reverse transcription polymerase chain reaction (RT-PCR; HR 2.57; 95% CI 1.49–4.45). Furthermore, high/positive ERCC1 expression was found to be associated with poor survival in patients receiving platinum-based chemotherapy in the RT-PCR group (HR 2.13; 95% CI 1.06–4.27). These data suggest that ERCC1 may be a useful prognostic factor for GC. In addition, low mRNA levels of ERCC1 appear to be associated with a significant favorable OS benefit from platinum-based chemotherapy.

## Introduction

Despite the fact that the incidence of gastric cancer (GC) has decreased substantially over the past few decades, it remains the fourth most common cancer and the second leading cause of cancer-related mortality worldwide (1). Generally, surgical resection with adjuvant chemotherapy is used to treat GC (2); however, the overall survival (OS) remains poor and no standard treatment has been determined. Discovering new molecular biological prognostic factors could provide a more accurate prediction of clinical outcome and help in the management of patients with GC.

Excision repair cross-complementing group 1 (ERCC1) protein, serving as a rate-limiting enzyme, is a key component in the nucleotide excision repair (NER) pathway (3). The NER pathway functions to remove bulky adducts that are introduced by platinum-containing drugs, such as cisplatin and oxaliplatin (4,5). Platinum-based chemotherapy for GC is one of the most widely used types of anticancer treatment (6,7). Previously, numerous studies have investigated the association between ERCC1 expression and survival in GC (8–10). In brief, they indicate that ERCC1 is not only a prognostic marker for survival but also a predictor of the response to platinum compounds; however the previous studies yielded inconsistent results and no robust evidence. Considering the potential value of ERCC1, a meta-analysis has been conducted to provide evidence-based results on the prognostic and predictive utility of ERCC1 in GC.

## Materials and methods

### Search strategy and inclusion criteria

A comprehensive search of Medline and Embase databases was conducted for all relevant literature published in the English language up to April 1, 2014. The medical subject headings for the search were ‘ERCC1’ and ‘gastric cancer’. In order to find additional studies, the references cited in the papers found in the database search were also manually searched. The eligible studies included in this meta-analysis met the following criteria: i) Patients with GC; ii) evaluation of ERCC1 expression and OS; and iii) presented the data for OS or data that allowed the OS to be calculated. When the patient population was duplicated, only the most recent or most complete study was included.

### Data extraction

Two authors independently reviewed all the potentially relevant studies and extracted the data required using standard forms. The following information was collected from the eligible studies: Surname of first author; year of publication; country; sample size; ERCC1 expression assessment method; cutoff values for high/positive vs*.* low/negative ERCC1 expression; stage; neoadjuvant or adjuvant chemotherapy; the hazard ratios (HRs) and their 95% confidence intervals (CIs). If HR was not directly reported, the HR estimate and its variance were reconstructed based on published methodology (11). Disagreements were resolved through discussion among the authors.

### Quality assessment

The study quality was evaluated by two investigators using the scale reported previously (Table I) (12,13). Briefly, this scale contained seven elements: i) The inclusion and exclusion criteria for patients; ii) the study design; iii) characteristics of the patients; iv) ERCC1 expression ascertainment method; v) the study endpoint; vi) follow-up time; and vii) time lost to follow-up. A high quality element was awarded one point and a maximum of two points was awarded for the method used to measure ERCC1 expression; hence, the maximum score was eight points. Each score provided by a different reader was compared and a consensus was achieved.

### Statistical analysis

The individual HRs corresponding to their 95% CIs were pooled into a summary HR to evaluate the association between ERCC1 level and OS. The significance of the summary HR was measured by a Z-test; P≤0.05 was considered to indicate statistical significance. Fixed-effects models were used with the assumption of homogeneity of studies in the first stage. The assumption was examined by assessing the heterogeneity across studies using χ^2^ test and I^2^. If the heterogeneity was significant between studies (P_heterogeneity_<0.1 and I^2^>50%), a random-effects model was performed in the second stage. Additionally, random-effects models were applied in cases where there was qualitative evidence of methodological heterogeneity within studies (e.g., different methods of measuring ERCC1 expression). To explore the possible heterogeneity among different studies, the following key characteristics were examined in a meta-regression model: Study location; ERCC1 expression ascertainment method; sample size; HR estimation method; and quality score. Sensitivity analysis was carried out by sequential omission of each study. Publication bias was evaluated with funnel plots, Begg’s test and Egger’s test (14). The analyses were performed with STATA software (version 12.0; Stata, College Station, TX, USA).

## Results

### Search results, study characteristics and quality assessment

**Figure 1:**
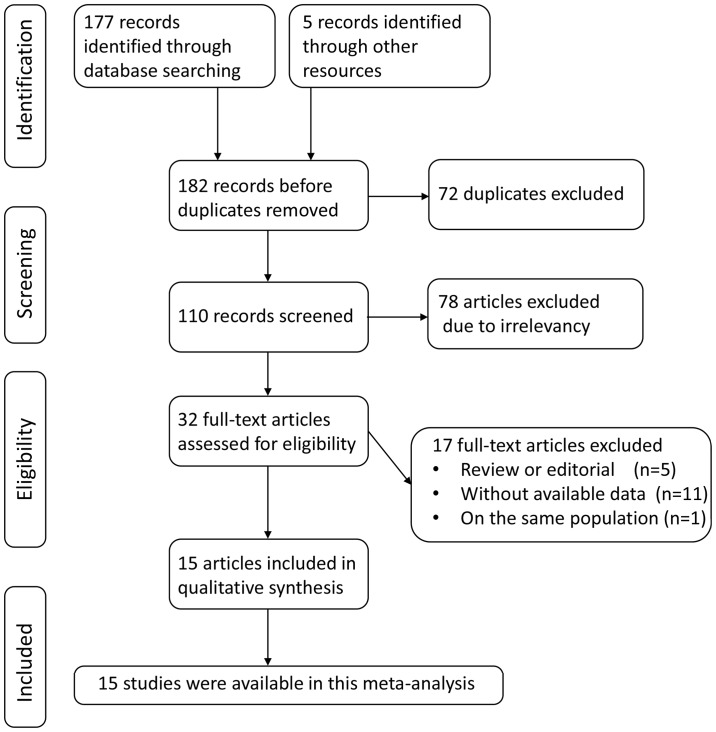
Flowchart of articles identified with criteria for inclusion and exclusion.

According to the search strategy, a total of 182 articles that mentioned ERCC1 expression and GC were identified. Following the removal of duplicates, 110 abstracts were screened based on the inclusion criteria. Among them, 32 articles remained for detailed evaluation. Seventeen of those 32 articles were subsequently excluded for the following reasons: Review or editorial (n=5); without available data (n=11); or on the same population (n=1). Finally, 15 studies were selected for this meta-analysis (8–10,15–26) (Fig. 1).

The main characteristics of the 15 studies are summarized in Table II. The sample size ranged from 41 to 322. Eleven studies were based in Asia, three in Europe and one in North America. The expression of ERCC1 was evaluated by immunohistochemistry (IHC) in ten studies, and reverse transcription polymerase chain reaction (RT-PCR) in five studies. Different cutoff values of ERCC1 expression evaluation were used. IHC was mainly divided by staining intensity and the percentage of cells stained, whereas ERCC1 mRNA levels were categorized according to median values and maximal χ^2^ method. In addition, the patients were receiving chemotherapy; most of them were using platinum-based regimens. The quality scores of included studies are summarized in Table II and ranged from 5 to 8.

### Quantitative synthesis

As shown in Table II, 10 of the 15 studies reported that high/positive ERCC1 expression in patients with GC was associated with poor survival, three studies indicated no association between ERCC1 expression and survival, and two studies exhibited an inverse association. Overall, the pooled HR for the 15 studies was 1.48 (95% CI 1.02–2.10; P=0.036; random-effects model) with significant heterogeneity (P_heterogeneity_<0.001, I^2^=83.8%), suggesting that high/positive ERCC1 expression was an indicator of poor survival in patients with GC.

**Figure 2:**
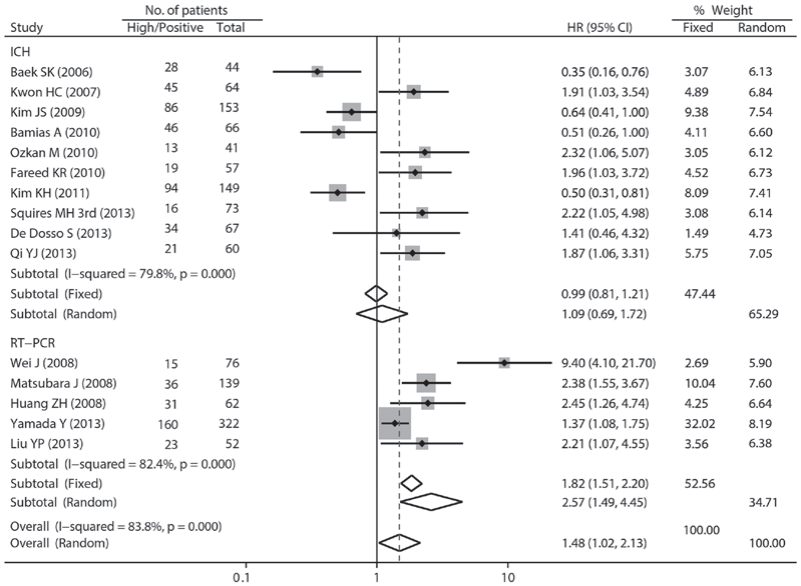
Pooled HRs for overall survival in patients with gastric cancer. The size of each square is proportional to the weight of the study (inverse of variance). HR, hazard ratio; CI, confidence interval; ICH, immunohistochemistry; RT-PCR, reverse transcription polymerase chain reaction.

When stratifying by study location, no association between ERCC1 expression and OS was observed in the Asian region (HR 1.54; 95% CI 0.99–2.38) or the non-Asian region (HR 1.31; 95% CI 0.63–2.75; Table III). Focusing the analysis on ERCC1 expression ascertainment methods, the pooled HR was 1.09 (95% CI 0.69–1.72) with an I^2^ of 79.8% for the ICH group, and 2.57 (95% CI 1.49–4.45) with an I^2^ of 82.4% for the RT-PCR group, respectively (Fig. 2). The significant correlation was also present in the subgroup analysis by sample size (<100), HR estimated (directly obtained) and quality score (7–8) (Table III).

**Figure 3:**
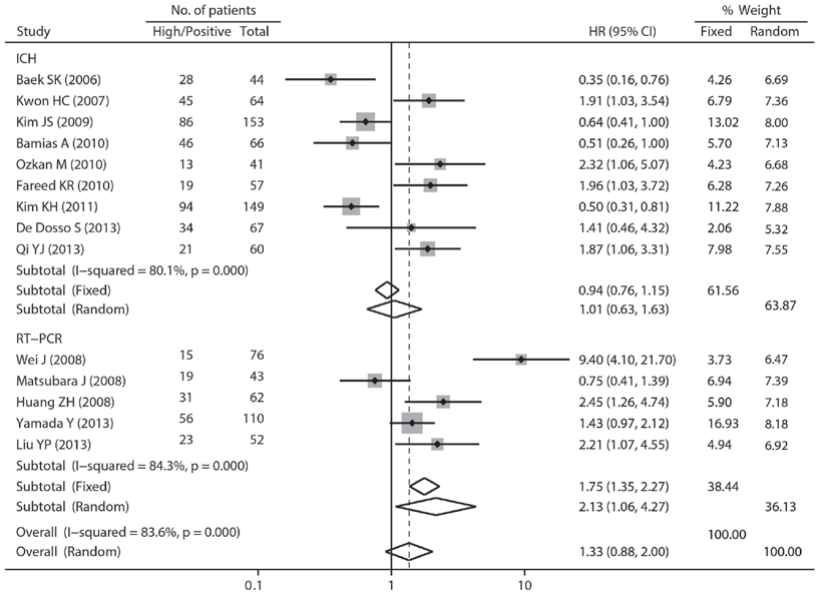
Pooled HRs for OS in GC patients receiving platinum-based chemotherapy. The size of each square is proportional to the weight of the study (inverse of variance). HR, hazard ratio; CI, confidence interval; OS, overall survival; GC, gastric cancer; ICH, immunohistochemistry; RT-PCR, reverse transcription polymerase chain reaction.

Of the 15 studies, there were 14 reports regarding the OS of patients receiving platinum-based chemotherapy. Meta-analysis of these studies also provided evidence of a trend toward poor survival with high ERCC1 expression (HR 1.33; 95% CI 0.88–2.00; I^2^=83.6%; random-effects model), although this was not considered statistically significant. In subgroup analysis by ERCC1 expression measurement method, a significant association was observed in the RT-PCR group (HR 2.13; 95% CI 1.06–4.27) with marked heterogeneity (I^2^=84.3%, Fig. 3).

### Meta-regression

In univariate meta-regression analysis, only the method used to measure ERCC1 expression (P=0.044) was found to be a significant source of heterogeneity (Table III); however, the estimated between-study variance (τ^2^) was reduced from 0.410 to 0.403, which could only explain 1.7% of the τ^2^.

### Sensitivity analysis and cumulative analysis

**Figure 4:**
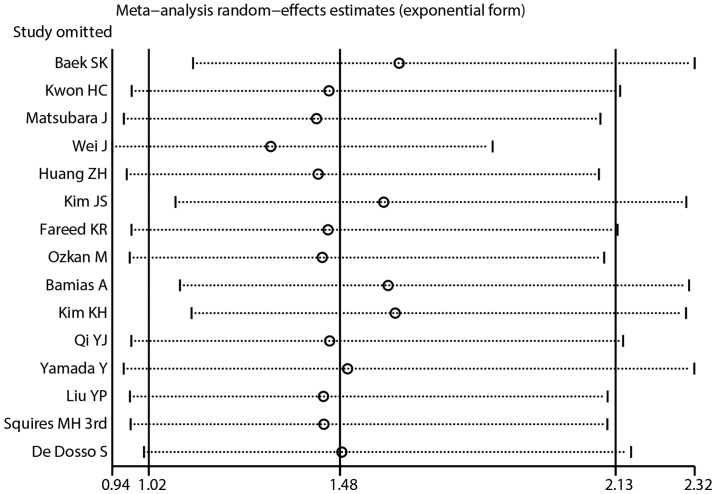
Influence analysis of the pooled hazard ratio for overall survival. Meta-analysis random effects estimates (exponential form) were used. Results were computed by omitting each study (on the left) in turn. The two ends of every broken line represented the 95% confidence interval.

**Figure 5:**
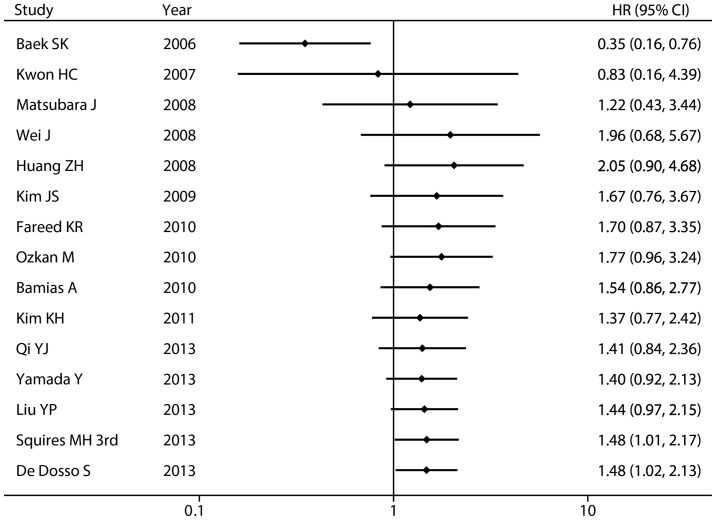
Cumulative meta-analysis showing the time-tendency of the HR for OS. HR, hazard ratio; CI, confidence interval, OS, overall survival.

Sensitivity analysis was performed to assess the influence of individual studies on the pooled HR. Similar HRs and 95% CIs were generated by omitting any single study using a random-effects model, and indicated that the results were relatively stable (Fig. 4). A cumulative meta-analysis of the 15 studies was conducted according to the publication date. As displayed in Fig. 5, the phenomenon that high ERCC1 expression was associated with a poor prognosis was first observed in the study reported by Squires *et al* (26) in 2013 (HR 1.48; 95% CI 1.01–2.17). Following that, only one study was added cumulatively, resulting in an overall effect estimate of 1.48 (95% CI 1.02–2.13).

### Publication bias

**Figure 6:**
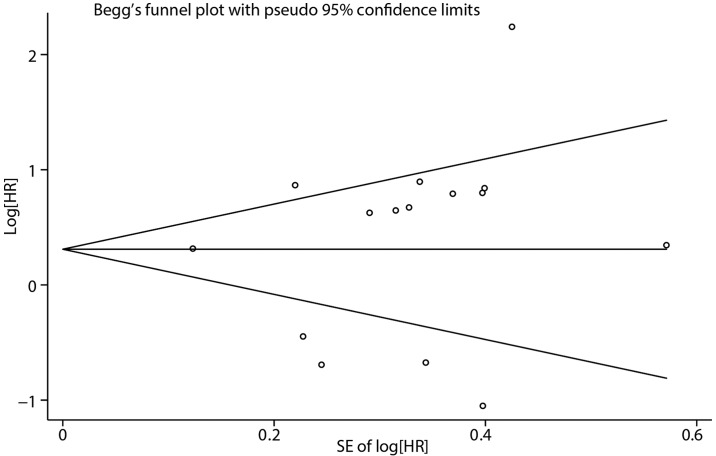
Funnel plot of the estimated publication bias of the included studies. HR, hazard ratio; SE, standard error.

The shape of the funnel plot did not exhibit any evident asymmetry (Fig. 6). The Begg’s and Egger’s tests indicated no evidence of publication bias (P=0.276 for Begg’s test; P=0.559 for Egger’s test).

## Discussion

The identification of molecular biomarkers with prognostic value for GC is clinically useful. In this meta-analysis, the effects of ERCC1 expression on the OS for GC were evaluated. The results indicate that high/positive ERCC1 expression is a significant poor prognostic factor for GC with chemotherapy regardless of the treatment regimen, compared with low/negative ERCC1 expression. Low mRNA levels of ERCC1 may be associated with a significant favorable OS benefit for platinum-based chemotherapy.

Numerous studies have reported that high/positive ERCC1 expression is associated with the prognosis of other types of cancer, including non-small cell lung (27), bladder (28), colorectal (29) and breast cancer (30). In addition, polymorphisms of ERCC1 have been found to affect OS in the platinum-based treatment of patients with GC (31). Overall, aberrant expression of ERCC1 appears to be associated with cancer risk. The biological role of ERCC1 may partly explain its poor prognosis. Cytotoxicity from platinum drugs leads to the formation of platinum DNA adducts, whereas ERCC1 acts to remove these bulky adducts and repair DNA double-strand damage. Furthermore, a high level of ERCC1 has been demonstrated to confer resistance to platinum agents and reconstitutes the ability of the cell to remove cisplatin from cellular DNA in an animal model (32). The aberrant methylation of DNA repair genes including ERCC1 has also been reported to affect the sensitivity to chemotherapeutic agents (33,34). The current results indicate that for the patients who received platinum-based chemotherapy, the risk of mortality increased with a high/positive expression of ERCC1 compared with the risk with low/negative ERCC1 expression.

To evaluate the effectiveness of different assessment methods, HRs were pooled from the IHC- or RT-PCR-based methods separately. In the present meta-analysis, RT-PCR appeared to be better than IHC in predicting OS for GC. ERCC1 expression using the IHC method was categorized by a visual grading system based on the staining intensity and percentage of cells stained, resulting in objectivity in certain circumstances. The ERCC1 mRNA levels were assessed with RT-PCR, which is a sensitive and quantitative method. This may one of the reasons why RT-PCR is more effective than IHC; however, the total sample size of the RT-PCR group was smaller than that of the IHC group (343 vs. 701). However, ERCC1 plays its role at the protein level. As is well-known, numerous factors can impact mRNA transcription. Notably, subgroup analyses demonstrated that the decreased survival associated with high ERCC1 expression was pronounced among high-quality-score studies. There is an urgent requirement for large-scale clinical studies to confirm these findings.

To the best of our knowledge, this was the first meta-analysis to evaluate the association between ERCC1 expression and the survival of patients with GC. There are, however, the following limitations to consider. Firstly, heterogeneity was significant in this meta-analysis. Although meta-regression analysis was used to clarify the source of heterogeneity, it was not successful. Additionally, sensitivity analysis did not help to find the source of heterogeneity. Secondly, where there were no directly obtained HRs in the studies, the estimated HRs were calculated from the data provided or extrapolated from the survival curves. The estimated HRs may be less reliable than those obtained directly from the papers. Thirdly, the cutoff values among these studies were different: Even in the IHC or RT-PCR subgroups the cutoff values were not unified. Studies with the same cutoff are, therefore, warranted to generate a more definitive conclusion. Fourthly, the cumulative meta-analysis presented significant associations until 2013, suggesting that this finding was not very robust with time. Finally, though no publication bias was detected in the present study, it could not be neglected. Since negative studies are often not published, and if these studies are published, they are often reported in a simplified way, this leads to difficulty in retrieving these data.

In conclusion, high/positive ERCC1 expression may be a poor prognostic factor for patients with GC. Due to the conferred resistance to platinum drugs, patients with high/positive ERCC1 expression (particularly with high ERCC1 mRNA levels) do not seem to benefit from platinum-based chemotherapy. Large scale and well-designed prospective studies are required to confirm the present findings.

## Figures and Tables

**Table I:** Criteria for quality assessment by De Graeff (12).

	Score	
	
Criteria	Sub-criteria	Criteria
1. Is the population under study defined with in and exclusion criteria?		1
2. Were patient data prospectively collected?		1
3. Are the main prognostic patient and tumor characteristics presented?^a^		1
4. Is the method used for determination of protein expression specified?		2
Criteria for immunohistochemistry:		
Is the immunohistochemical staining protocol specified?^b^	1	
Were stainings evaluated by >1 observer?	1	
Criteria for RT-PCR:		
Is the RNA isolation method and cDNA synthesis specified?	1	
Is the PCR protocol specified?^c^	1	
5. Is the study endpoint defined?		1
6. Is the time of follow up specified?		1
7. Is loss during analysis or follow up described?		1
Total		8

a At least four of the following characteristics: age at diagnosis, tumor stage, tumor location, differentiation grade and residual tumor after primary surgery.

b At least four of the following criteria: antigen retrieval, primary antibody, dilution, detection method, cut-off value for positive expression:

c At least the primers used and the annealing temperature or number of cycles.

**Table II:** Main characteristics of all studies included in this meta-analysis.

First author (ref)	Year	Location	No. of patients		Method	Cutoff	Stage	Chemotherapy	Survival analysis	Quality score

			High/Positive	Total						
Baek (15)	2006	Korea	28	44	ICH	Percentage of cells stained 10%	Advanced	Cisplatin, 5-FU	Estimated	5
Kwon (22)	2007	Korea	45	64	ICH	Both staining intensity and percentage of cells stained ≥2	Advanced	Oxaliplatin, 5-FU	Multivariate	7
Wei (9)	2008	China	15	76	RT-PCR	Maximal χ^2^ method 0.47	III–IV	Oxaliplatin, 5-FU	Multivariate	7
Matsubara (10)	2008	Japan	36	139	RT-PCR	Maximal χ^2^ method 1.42×10^−3^	Advanced	Cisplatin, S-1	Univariate	7
Huang (19)	2008	China	31	62	RT-PCR	Median 0.672	I–IV	Oxaliplatin, 5-FU	Multivariate	7
Kim (20)	2009	Korea	86	153	ICH	Staining <17.5 vs. ≥17.5	III–IV	Cisplatin, 5-FU	Univariate	5
Bamias (16)	2010	Greece	46	66	ICH	Staining 0–1 vs. 2–6	I–IV	Cisplatin/carboplatin, docetaxel	Univariate	8
Ozkan (24)	2010	Turkey	13	41	ICH	Percentage of cells stained 10%	Advanced	Cisplatin, 5-FU	Estimated	6
Fareed (18)	2010	UK	19	57	ICH	Staining 0 vs. 1–3	I–IV	Cisplatin, 5-FU/Xeloda	Estimated	6
Kim (21)	2011	Korea	94	149	ICH	Both staining intensity and percentage of cells stained ≥2	II–IV	Cisplatin, 5-FU	Estimated	6
Squires (26)	2013	USA	16	73	ICH	Staining 0–2 vs. 3–4	I–III	5-FU, radiation	Multivariate	6
De Dosso (17)	2013	Switzerland	34	67	ICH	Staining 0–1 vs. 2–3	II–III	Cisplatin, 5-FU	Univariate	5
Yamada (8)	2013	Japan	160	322	RT-PCR	Median	Advanced	Cisplatin + irinotecan, 5-FU, S-1	Univariate	8
Liu (23)	2013	China	23	52	RT-PCR	Median 7.32	I–IV	Oxaliplatin/cisplatin	Multivariate	7
Qi (25)	2013	China	21	60	ICH	Median value of multiplying staining intensity by percentage of cells stained	Advanced	Oxaliplatin, 5-FU	Estimated	5

RT-PCR, reverse transcriptase polymerase chain reaction; IHC, immunohistochemistry; 5-FU, 5-fluorouracil; S-1, tegafur.

**Table III:** Stratified analysis of excision repair cross-complementation group 1 expression with overall survival.

Variables	n^a^	Pooled HR (95% CI)		Heterogeneity test			Meta-regression P-value
	
		Fixed	Random	Q	P-value^b^	I^2^ (%)	
Location							0.759
Asian	11	1.38 (1.19, 1.59)	1.54 (0.99, 2.38)	75.37	<0.001	86.7	
Non-Asian	4	1.28 (0.88, 1.86)	1.31 (0.63, 2.75)	10.81	0.013	72.3	
Method							0.044
IHC	10	0.99 (0.82, 1.21)	1.09 (0.69, 1.72)	44.58	<0.001	79.8	
RT-RCR	5	1.82 (1.51, 2.20)	2.57 (1.49, 4.45)	22.78	<0.001	82.4	
Sample size							0.262
<100	11	1.72 (1.39, 2.14)	1.74 (1.08, 2.80)	47.53	<0.001	79.0	
≥100	4	1.16 (0.97, 1.39)	1.02 (0.55, 1.91)	31.14	<0.001	90.4	
HR estimated							0.304
Directly obtained	10	1.50 (1.28, 1.76)	1.73 (1.13, 2.64)	52.25	<0.001	82.8	
Indirectly obtained	5	1.02 (0.77, 1.34)	1.08 (0.51, 2.29)	28.27	<0.001	85.9	
Quality score							0.177
5–6	8	0.98 (0.78, 1.22)	1.12 (0.66, 1.89)	36.49	<0.001	80.8	
7–8	7	1.68 (1.41, 2.00)	1.99 (1.22, 3.26)	35.66	<0.001	83.2	

a Number of comparisons.

b P-value of Q-test for heterogeneity test.

IHC, immunohistochemistry; RT-PCR, reverse transcription-polymerase chain reaction; HR, hazard ratio.
